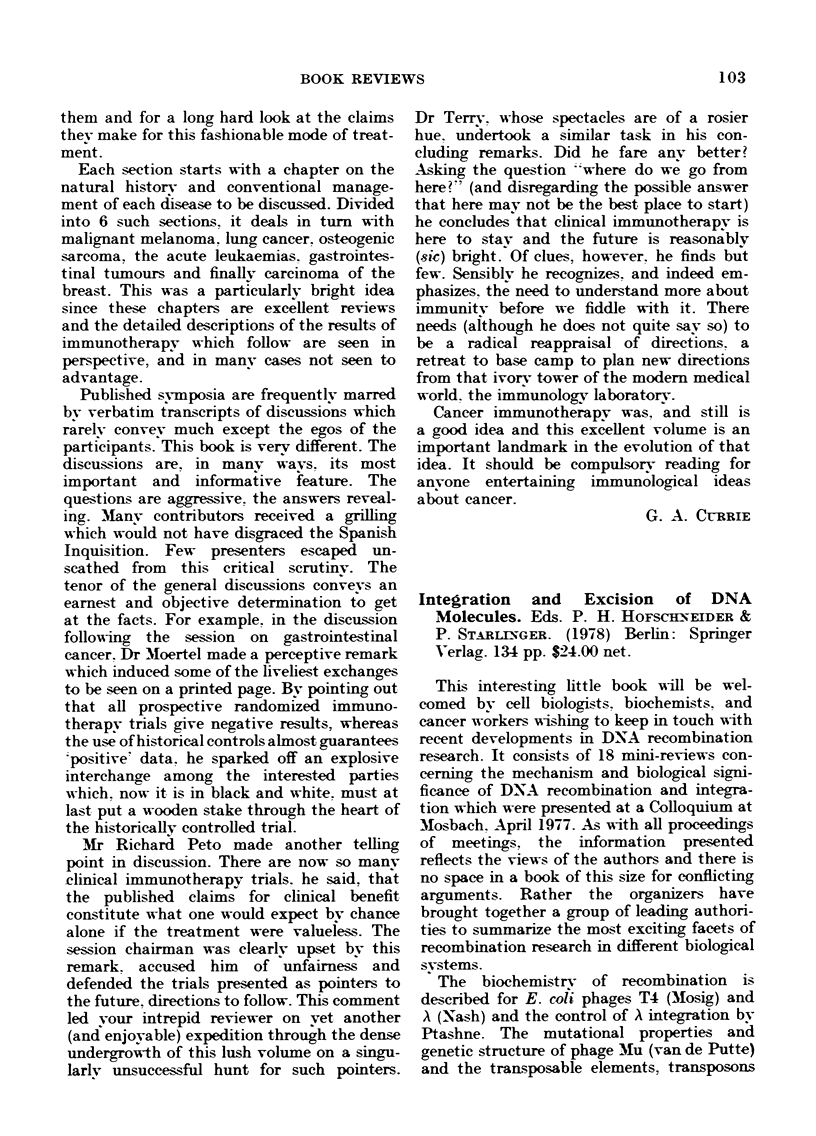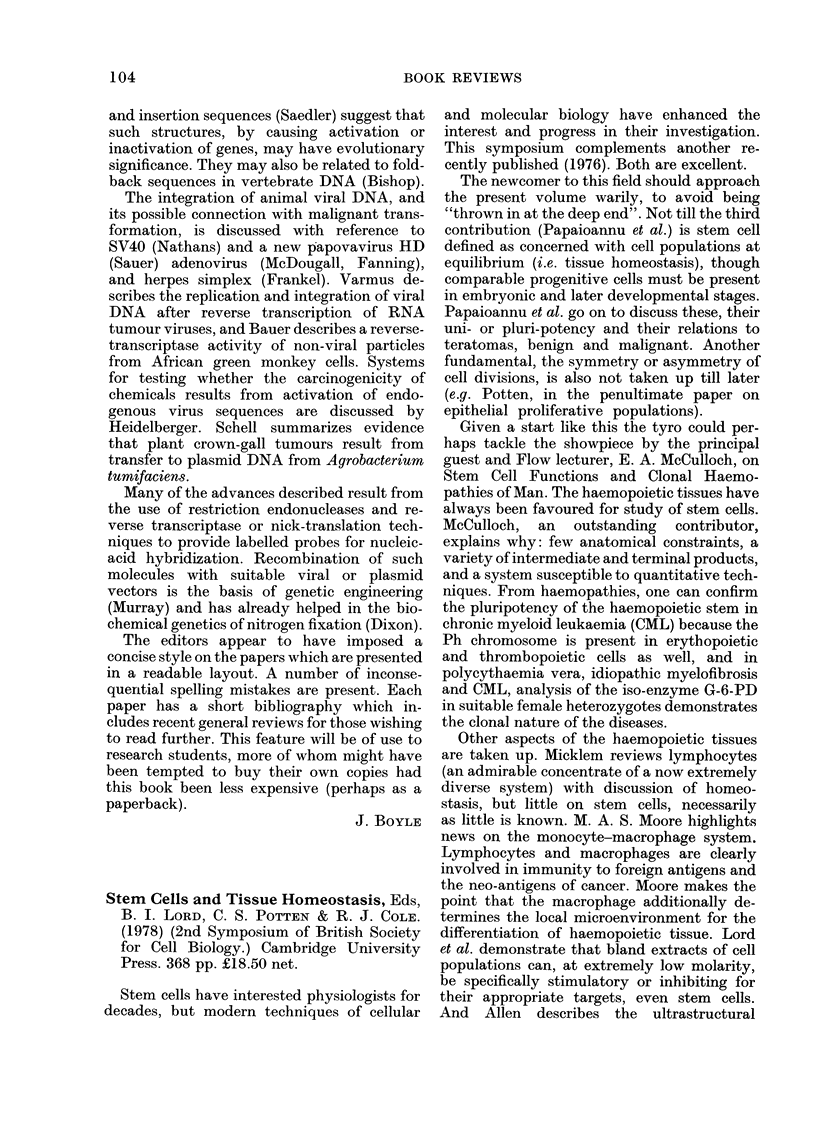# Integration and Excision of DNA Molecules

**Published:** 1979-01

**Authors:** J. Boyle


**Integration and Excision of DNA Molecules.** Eds. P. H. Hofschneider & P. Starlinger. (1978) Berlin: Springer Verlag. 134 pp. $24.00 net.

This interesting little book will be welcomed by cell biologists, biochemists, and cancer workers wishing to keep in touch with recent developments in DNA recombination research. It consists of 18 mini-reviews concerning the mechanism and biological significance of DNA recombination and integration which were presented at a Colloquium at Mosbach, April 1977. As with all proceedings of meetings, the information presented reflects the views of the authors and there is no space in a book of this size for conflicting arguments. Rather the organizers have brought together a group of leading authorities to summarize the most exciting facets of recombination research in different biological systems.

The biochemistry of recombination is described for *E. coli* phages T4 (Mosig) and $\lambda$ (Nash) and the control of $\lambda$ integration by Ptashne. The mutational properties and genetic structure of phage Mu (van de Putte) and the transposable elements, transposons and insertion sequences (Saedler) suggest that such structures, by causing activation or inactivation of genes, may have evolutionary significance. They may also be related to fold-back sequences in vertebrate DNA (Bishop).

The integration of animal viral DNA, and its possible connection with malignant transformation, is discussed with reference to SV40 (Nathans) and a new papovavirus HD (Sauer) adenovirus (McDougall, Fanning), and herpes simplex (Frankel). Varmus describes the replication and integration of viral DNA after reverse transcription of RNA tumour viruses, and Bauer describes a reverse-transcriptase activity of non-viral particles from African green monkey cells. Systems for testing whether the carcinogenicity of chemicals results from activation of endogenous virus sequences are discussed by Heidelberger. Schell summarizes evidence that plant crown-gall tumours result from transfer to plasmid DNA from *Agrobacterium tumifaciens*.

Many of the advances described result from the use of restriction endonucleases and reverse transcriptase or nick-translation techniques to provide labelled probes for nucleic-acid hybridization. Recombination of such molecules with suitable viral or plasmid vectors is the basis of genetic engineering (Murray) and has already helped in the biochemical genetics of nitrogen fixation (Dixon).

The editors appear to have imposed a concise style on the papers which are presented in a readable layout. A number of inconsequential spelling mistakes are present. Each paper has a short bibliography which includes recent general reviews for those wishing to read further. This feature will be of use to research students, more of whom might have been tempted to buy their own copies had this book been less expensive (perhaps as a paperback).

J. Boyle